# Anti-Infective Potential of Marine Invertebrates and Seaweeds from the Brazilian Coast

**DOI:** 10.3390/molecules18055761

**Published:** 2013-05-16

**Authors:** Éverson Miguel Bianco, Simone Quintana de Oliveira, Caroline Rigotto, Maiko Luis Tonini, Tatiana da Rosa Guimarães, Francine Bittencourt, Lidiane Pires Gouvêa, Cassandra Aresi, Maria Tereza Rojo de Almeida, Maria Izabel Goularte Moritz, Cintia Dalcuche Leal Martins, Fernando Scherner, João Luís Carraro, Paulo Antunes Horta, Flávio Henrique Reginatto, Mario Steindel, Cláudia Maria Oliveira Simões, Eloir Paulo Schenkel

**Affiliations:** 1 Laboratório de Produtos Naturais, Departamento de Ciências Farmacêuticas, Universidade Federal de Santa Catarina, CEP 88.040-900, Florianópolis, SC, Brasil; E-Mails: ebianco@chemist.com (E.M.B.); simonequintana@hotmail.com (S.Q.d.O.); tatyguimaraes@gmail.com (M.T.d.R.G.); francine-fb@hotmail.com (F.B.); cassandraaresi@hotmail.com (C.A.); terezarojo@gmail.com (M.T.R.d.A.); mizabelgm@gmail.com (M.I.G.M.); freginatto@hotmail.com (F.H.R.); 2 Laboratório de Ficologia, Departamento de Botânica, Universidade Federal de Santa Catarina, CEP 88.040-900, Florianópolis, SC, Brasil; E-Mails: lidi_pel@hotmail.com (L.P.G.); cintia_ufpr@yahoo.com.br (C.D.L.M.); fscherner@yahoo.com (F.S.); pahorta@hotmail.com.br (P.A.H.); 3 Laboratório de Virologia Aplicada, Departamento de Ciências Farmacêuticas, Universidade Federal de Santa Catarina, CEP 88.040-900, Florianópolis, SC, Brasil; E-Mails: rigottocarol@gmail.com (C.R.); claudias@reitoria.ufsc.br (C.M.O.S.); 4 Laboratório de Protozoologia, Departamento de Microbiologia, Imunologia e Parasitologia, Universidade Federal de Santa Catarina, CEP 88.040-900, Florianópolis, SC, Brasil; E-Mails: maikotonini@hotmail.com (M.L.T.); mario.steindel@ufsc.br (M.S); 5 Programa de Pós-graduação em Ecologia, Universidade Federal do Rio Grande do Sul, CEP 91.501-970, Porto Alegre, RS, Brasil; E-Mail: joao.porifera@gmail.com

**Keywords:** marine natural products, seaweeds, marine invertebrates, antileishmanial activity, antitrypanosomal activity, antimicrobial activity, anti-HSV-1 activity

## Abstract

This manuscript describes the evaluation of anti-infective potential *in vitro* of organic extracts from nine sponges, one ascidian, two octocorals, one bryozoan, and 27 seaweed species collected along the Brazilian coast. Antimicrobial activity was tested against *Staphylococcus aureus* (ATCC 25923), *Enterococcus faecalis* (ATCC 29212), *Pseudomonas aeruginosa* (ATCC 27853), *Escherichia coli* (ATCC 25922) and *Candida albicans* (ATCC 10231) by the disk diffusion method. Antiprotozoal activity was evaluated against *Leishmania braziliensis* (MHOM/BR/96/LSC96-H3) promastigotes and *Trypanosoma cruzi* (MHOM/BR/00/Y) epimastigotes by MTT assay. Activity against intracellular amastigotes of *T. cruzi* and *L. brasiliensis* in murine macrophages was also evaluated. Antiviral activity was tested against Herpes Simplex Virus type 1 (HSV-1, KOS strain) by the plaque number reduction assay (IC_50_). Cytotoxicity on VERO cells was evaluated by the MTT assay (CC_50_). The results were expressed as SI = CC_50_/IC_50_. The most promising antimicrobial results were obtained against *S. aureus* and *C. albicans* with *Dragmacidon reticulatum*. Among the seaweeds, only *Osmundaria obtusiloba* showed moderate activity against *P. aeruginosa*. Concerning antiprotozoal activity, *Bugula neritina*, *Carijoa riseii*, *Dragmaxia anomala* and *Haliclona* (*Halichoclona*) sp. showed the most interesting results, mainly against extracellular promastigote forms of *L. braziliensis* (66, 35.9, 97.2, and 43.6% inhibition, respectively). Moreover, six species of seaweeds *Anadyomene saldanhae*, *Caulerpa cupressoides*, *Canistrocarpus cervicornis*, *Dictyota* sp., *Ochtodes secundiramea*, and *Padina* sp. showed promising results against *L. braziliensis* (87.9, 51.7, 85.9, 93.3, 99.7, and 80.9% inhibition, respectively), and only *Dictyota* sp. was effective against *T. cruzi* (60.4% inhibition). Finally, the antiherpes activity was also evaluated, with *Haliclona* (*Halichoclona*) sp. and *Petromica citrina* showing the best results (SI = 11.9 and SI > 5, respectively). All the active extracts deserve special attention in further studies to chemically characterize the bioactive compounds, and to perform more refined biological assays.

## 1. Introduction

Marine natural products represent an immeasurable potential source of new drugs with diverse and often unique structures [1], and diverse biological properties, such as antiviral [2], antibacterial [3], antiprotozoal [4,5,6], antifungal [7], cytotoxic [8,9,10] and antitumoral activities [11,12] have been reported. Success in these areas is demonstrated by several new compounds in pre- or clinical evaluation [13,14].

Brazil is a continental country, with 8,500 km of Atlantic coastline that supports an exclusive and rich diversity of endemic marine fauna and flora that can offer rich rewards for the chemical study of marine natural products in the search for novel bioactive secondary metabolites with potential medicinal properties. However, so far only a few classes of Brazilian marine organisms have been investigated for their chemical and pharmacological properties [15,16,17,18,19,20,21,22,23,24,25]. We therefore believe that the identification of Brazilian organisms with significant biotechnological potential for use in drugs is an important goal [18].

Some studies regarding bioprospection of Brazilian marine organisms have been reported. In 2002, Monks and co-workers performed the first biological screening with marine sponges collected from the Santa Catarina coast, in the south of Brazil. Several activities, such as cytotoxic, antichemotactic and antimicrobial properties were detected for the organic and aqueous extracts of 10 marine sponges [19].

Silva *et al.* [20] evaluated the *in vitro* antiherpes (HSV-1, KOS strain), anti-adenovirus (human AdV serotype 5) and anti-rotavirus (simian RV SA11) activities of extracts from 27 different marine sponges (Porifera) collected from the Brazilian coast. The results showed that the aqueous extracts from *Cliona* sp., *Agelas* sp., *Tethya* sp., *Axinella* aff. *corrugata*, *Polymastia janeirensis* and *Protosuberites* sp. were highly promising and deserve special attention in further studies. Furthermore, Frota-Jr and co-authors reported the antitumor activity of the marine sponge *P. janeirensis* in human U138MG glioma cell line [21,22].

Jimenez and colleagues performed the first ascidian antitumor screening with organisms from the Northeast coast of Brazil. The results suggest these are a rich source of natural compounds with cytotoxic properties [23].

Seleghim *et al.* screened 349 crude extracts from marine sponges, ascidians, bryozoans, and octocorals collected along the Brazilian coastal against bacteria strains, yeasts, *Mycobacterium tuberculosis*, cancer cell lines [MCF-7 (breast cancer), B16 (murine melanoma) and HCT8 (colon)]. The results showed a high percentage of bioactive extracts from the phyla Porifera, Ascidiacea, Cnidaria and Bryozoa [24].

Recently, Soares and colleagues [25] evaluated the antiviral activity of extracts from 36 species of seaweeds from seven locations of the Brazilian coastline against HSV-1 and HSV-2 strains. The results obtained reinforce the role of seaweeds as an important source of compounds with for the development of new drugs against herpes.

The marine biodiversity loss that has been observed worldwide [26], but especially in Brazil, is driving an unprecedented loss of biotechnological potential related with these organisms [27,28]. In attention to the human constant need for new drugs and therapies in the present work, we performed an anti-infective (antibacterial, antifungal, antiprotozoal and antiviral) screening of 95 different extracts and fractions from 13 marine invertebrates collected from the southern Brazilian coast, and 27 seaweeds from the northeastern Brazilian coast.

## 2. Results and Discussion

This paper describes the *in vitro* antimicrobial, antiprotozoal and antiviral evaluation of organic extracts and fractions from 13 marine invertebrate species (nine sponges, one ascidian, two octocorals, and one bryozoans (Table 1), and 27 seaweeds species [sixteen Rhodophyta (59.2%), seven Phaeophyceae (26%), and five Chlorophyta (14.8%) (Table 2). A total of 95 extracts and fractions (65 from marine invertebrates and 30 from seaweeds) were assayed. The results showed that 53 samples (56%) exhibited some anti-infective activity against *Staphylococcus aureus*, *Enterococcus faecalis*, *Pseudomonas aeruginosa*, *Escherichia coli* and *Candida albicans* (antimicrobial), *Leishmania braziliensis* and *Trypanosoma cruzi* (anti-protozoal), as well as against HSV-1 replication (antiviral).

**Table 1:** Marine invertebrates collected for biological assays.

Species	Collection local and deep	Collection date
Phylum Cnidaria (Octocorallia)		
* Carijoa riisei*	Xavier Island (10–14 m deep)	May 2011
* Leptogorgia punicea*	Aranhas Island (10–14 m deep)	April 2011
Phylum Bryozoa		
* Bugula neritina*	Sambaqui Beach (1–2 m deep)	October 2011
Phylum Porifera		
Cliona celata	Xavier Island (10–12 m deep)	March 2011
* Dragmacidon reticulatum*	Xavier Island (10–14 m deep)	May 2011
* Dragmaxia anomala*	Aranhas Island (10–14 m deep)	December 2011
* Guitarra sepia*	Xavier Island (7–14 m deep)	May 2011
* Haliclona* (*Halichoclona*) sp.	Aranhas Island (10–14 m deep)	April 2011
* Petromica citrina*	Xavier Island (9–17 m deep)	January–July 2010
* Polymastia janeirensis*	Xavier Island (10–14 m deep)	December 2011
* Tedania ignis*	Aranhas Island (6–10 m deep)	April 2011
* Trachycladus* sp.	Campeche Island (15 m deep)	May 2011
Phylum Urochordata (Tunicate)		
* Didemnum granulatum*	Aranhas Island (7–14 m deep)	April 2011

**Table 2:** Marine seaweeds collected for biological assays.

Species	Collection local and deep ^#^	Collection date
Phylum Rhodophyta		
* Acanthophora specifera*	Conceição Lagoon, SC (27°36'29'' S; 48°26'31'' W)	March 2012
* Botryocladia occidentalis*	Taíba Beach, CE (03°30'27'' S; 38°55'11'' W)	August 2011
* Bryothamnion seaforthii*	Taíba Beach, CE (03°30'27'' S; 38°55'11'' W)	August 2011
* Bryothamnion triquetrum*	Taíba Beach, CE (03°30'27'' S; 38°55'11'' W)	August 2011
* Bryothamnion triquetrum*	Farol de Itapoã Beach, BA (12°57'25'' S; 38°21'15'' W)	September 2011
* Cryptonemia seminervis*	Taíba Beach, CE (03°30'27'' S; 38°55'11'' W)	August 2011
* Digenea simplex*	Atol das Rocas, RN (03° 51'03'' S, 33° 40'29'' W)	February 2012
* Gracilaria caudate*	Taíba Beach, CE (03°30'27'' S; 38°55'11'' W)	August 2011
* Gracilaria cervicornis*	Taíba Beach, CE (03°30'27'' S; 38°55'11'' W)	August 2011
* Gracilaria cervicornis*	Arraial d´Ajuda Beach, BA (16°29'54'' S; 39° 04'07'' W)	September 2011
* Grateloupia cuneifolia*	Canasvieiras Beach, SC (27°25'29'' S; 48°26'43'' W)	October 2011
* Hypnea cenomyce*	Taíba Beach, CE (03°30'27'' S; 38°55'11'' W)	August 2011
* Hypnea musciformis*	Taíba Beach, CE (03°30'27'' S; 38°55'11'' W)	August 2011
* Laurencia dendroidea*	Arraial d´Ajuda Beach, BA (16°29'54'' S; 39° 04'07'' W)	September 2011
* Ochtodes secundiramea*	Arraial d´Ajuda Beach, BA (16°29'54'' S; 39° 04'07'' W)	September 2011
* Osmundaria obtusiloba*	Cabo Branco Beach, PB (07°07'31'' S; 34°49'19'' W)	July 2012
* Palisada flagellifera*	Enseada dos Corais Beach, PE (08°19'23'' S; 34° 56'55'' W)	March 2012
* Palisada papillosa*	Arraial d´Ajuda Beach, BA (16°29'54'' S; 39° 04'07'' W)	September 2011
Class Pheophyceae		
* Canistrocarpus cervicornis*	Arraial d´Ajuda Beach, BA (16°29'54'' S; 39° 04'07'' W)	September 2011
* Dictyopteris delicatula*	Da Barra Beach, BA (16°29'54'' S; 39° 04'07'' W)	September 2011
* Dictyopteris jolyana*	Cabo Branco Beach, PB (07°07'31'' S; 34°49'19'' W)	July 2012
* Dictyota* sp.	Arraial d´Ajuda Beach, BA (16°29'54'' S; 39° 04'07'' W)	March 2012
* Padina* sp.	Farol de Itapoã Beach (12°57'25'' S; 38°21'15'' W)	September 2011
* Padina gymnospora*	Arraial d´Ajuda Beach, BA (16°29'54'' S; 39° 04'07'' W)	March 2012
* Sargassum* sp.	Arraial d´Ajuda Beach, BA (16°29'54'' S; 39° 04'07'' W)	September 2011
Phylum Chlorophyta		
* Anadyomene saldanhae*	Arraial d´Ajuda Beach, BA (16°29'54'' S; 39° 04'07'' W)	September 2011
* Anadyomene stellata*	Arraial d´Ajuda Beach, BA (16°29'54'' S; 39° 04'07'' W)	May 2012
* Caulerpa sertularioides*	Arraial d´Ajuda Beach, BA (16°29'54'' S; 39° 04'07'' W)	September 2011
* Caulerpa* cupressoides ^a^	Farol da Barra Beach, BA (13°00'40'' S 38°31'55'' W)	September 2011
* Caulerpa cupressoides* ^b^	Arraial d´Ajuda Beach, BA (16°29'54'' S; 39° 04'07'' W)	September 2011

*#* *All seaweeds were collected in the intertidal zone. ^a,b^ same species, but collected in different locales.*

### 2.1. Marine Invertebrates

Thirteen species of marine invertebrates were assayed against bacteria and fungus. Of these, seven [*Dragmacidon reticulatum*, *Dragmaxia anomala*, *Haliclona* (*Halichoclona*) sp., *Leptogorgia punicea*, *Petromica citrina*, *Tedania ignis*, and *Trachycladus* sp.], showed some activity (Table 3).

The most interesting antimicrobial results were obtained with the sponge *D. reticulatum* thatshowed significant growth inhibition (13–16 mm) against *S. aureus* and *C. albicans*. A similar result was proved in another screening carried out with marine organisms from the southeastern Brazilian coast where the sponge *D. reticulatum* showed a weak antimicrobial activity against *S. aureus* and *C. albicans* [24]*.* As far as we are aware, this is the first report of this biological activity for *D. reticulatum*, *D. anomala*, and *Trachycladus* sp.

Furthermore, a weak antimicrobial activity against *S. aureus*, *E. faecalis*, and *E. coli* was also detected in the present study for *Haliclona* (*Halichoclona*) sp. and *Petromica citrina* (9–12 mm inhibition zone), and *T. ignis* (6–8 mm inhibition zone). In the same way, we verified a weak antimicrobial activity against *S. aureus* and *C. albicans* of the *n*-hexane extract from the octocoral *Leptogorgia punicea*.

**Table 3:** Antibacterial and antifungal screening of marine invertebrates by disc diffusion method.

Species	Extracts	Bacterial and fungal strains				
		*S. aureus*	*E. faecalis*	*E. coli*	*P. aeruginosa*	*C. albicans*
*Dragmaxia anomala*	E1	+	+	−	−	−
*Dragmacidon reticulatum*	E3F1	+++	−	−	−	+++
*Haliclona (Halichoclona)* sp.	E3F2	++	++	+	-	+
*Leptogorgia punicea*	E1	++	−	−	−	++
*Petromica citrina*	E3F2	++	++	-	-	++
*Tedania ignis*	E2	−	+	−	−	−
*Trachycladus* sp.	E3F2	++	++	+	−	+
	E3F3	−	+	−	−	−

(−): no activity; (+): 6–8 mm of inhibition zone; (++): 9–12 mm of inhibition zone; (+++): 13–16 mm of inhibition zone. Positive controls: *S. aureus*: oxacillin (1 µg) 18–24 mm; *E. faecalis*: ampicillin (10 µg) > 17 mm; *P. aeruginosa*: ceftazidime (30 µg) 22–29 mm; *E. coli*: ampicillin (10 µg) 16–22 mm; *C. albicans*: fluconazole (25 µg) > 19 mm; E1: *n*-hexane extract; E2: dichloromethane extract; E3F1: ethyl acetate fraction from E3 (methanol extract); E3F2: *n*-butanol fraction from E3 (methanol extract); E3F3: aqueous residue from E3 (methanol extract).

As far as we aware, this is the first report for antimicrobial activity for this gorgonian species. On the other hand, the extracts from *B. neritina*, *C. riseii*, *C. celata*, *D. granulatum*, *G. sepia* and *P. janeirensis* did not show antimicrobial activity against the assayed microorganism strains.

Recently it was reported that aqueous extract of *P. citrina* (collected in Rio de Janeiro State, southeast of Brazil) showed a large spectrum of activity against clinical strains and resistant-bacteria including *S. aureus*, *S. epidermidis*, *E. coli*, *E. faecalis*, *M. fortuitum* and *N. gonorrhoeae.* All these activities were related to the presence of halistanol trisulphate A in this marine sponge [29,30]. Moreover, the antifungal activity for this substance, isolated from *Petromica ciocalyptoides* was also reported [31].

Another study led by Monks *et al.* [19] concerning the antimicrobial activity against *E. coli*, *S. aureus*, *S. epidermis*, *B. subtilis*, and *M. luteus* strains of southern Brazilian sponges, including *Guitarra* sp., *T. ignis*, *Haliclona* aff. *tubifera*, demonstrated that *H*. aff. *tubifera* showed moderate activity against *E. coli* and weak activity against *S. aureus*, *S. epidermis*,and *M. luteus*. Our results are in agreement with those obtained by these authors, although we used different libraries of microbial strains and extracts.

Concerning antiprotozoal activity, few studies reporting antileishmanial and tripanocidal activities have been described for marine invertebrates. In this work, 13 marine invertebrate species were evaluated against *L. brasiliensis* and *T. cruzi* (Table 4).

**Table 4:** Antiprotozoal activity expressed as growth inhibition (%) of extracts and fractions obtained from marine invertebrates.

Species	Samples	*Leishmania braziliensis* (promastigotes)	*Trypanosoma cruzi* (epimastigotes)
*Bugula neritina*	E1	66	−
	E3F2	47	−
	E3F3	30.7	−
*Carijoa riisei*	E1	35.9	43.4
	E2	−	29
	E3F1	−	26.1
	E3F2	14.6	2.6
	E3F3	−	5.5
*Didemnun granulatum*	E1	−	21.5
	E3F1	15.7	13.2
	E3F2	17.9	−
*Dragmacidon reticulatum*	E1	24.1	11.4
	E2	−	20
	E3F1	−	21.2
	E3F2	19.8	−
	E3F3	13.8	15.3
*Dragmaxia anomala*	E1	97.2	71.7
*Guitarra sepia*	E3F2	12.5	−
	E3F3	14.9	−
*Haliclona (Halichoclona) *sp.	E1	−	14.6
	E3F1	−	28.3
	E3F2	43.6	33
	E3F3	16.9	−
*Leptogorgia punicea*	E1	−	19.2
	E2	−	38.8
	E3F2	−	11.1
	E3F3	15	−
*Tedania ignis*	E2	16.1	−
	E3F1	12.2	−
	E3F2	18.4	−
	E3F3	19.1	−

Extracts and fractions concentration: 50 µg/mL; (−): no activity; E1: hexane extract; E2: dichlorometane extract; E3F1: ethyl acetate fraction from E3 (methanol extract); E3F2: *n*-butanol fraction from E3 (methanol extract); E3F3: aqueous residue from E3 (methanol extract).

Out of these 13 species tested, *Bugula neritina* (E1 extract), *Carijoa riisei* (E1 extract), *Dragmaxia anomala* (extract E1), and *Haliclona* (*Halichoclona*) sp. (fraction E3F2) showed the best results, particularly against extracellular promastigote forms of *L. braziliensis* (66, 35.9, 97.2 and 43.6% grown inhibition, respectively). However, only two species, *C. riisei* and *D. anomala* showed some tripanocidal effects (43.4 and 71.7% growth inhibition, respectively). 

Additionally, these extracts and fractions were assayed on *L. brasiliensis* amastigotes in bone marrow macrophages from mice, and only the sponge *Haliclona* (*Halichoclona*) sp. and the octocoral *C. riisei* were active (Table 5). Based on these preliminary results, the E1 extract from *C. riisei* was fractionated by chromatographic techniques leading to the isolation of an active pregnane steroid [32]. Finally, the extracts from *C. celata*, *P. citrina*, *P. janeirensis* and *Trachycladus* sp. were not active against *L. braziliensis* or *T. cruzi*.

**Table 5:** Effects of marine invertebrates extracts and fractions on *Leishmania brasiliensis* amastigotes in bone marrow macrophages from mice, and cytotoxicity on J774.G8 macrophage cell line.

Species	Samples	CC_50_ ± SD (µg/mL)	IC_50_ ± SD (µg/mL)	Selective index (CC_50_/IC_50_)
*Bugula neritina*	E1	ND	>50	ND
*Carijoa riisei*	E1	48.6 ± 4.8	43.3 ± 8.5	1.1
*Dragmaxia anomala*	E1	54.3 ± 1.9	>15	<3.6
*Haliclona (Halichoclona)* sp.	E3F2	279.7 ± 21.2	43.9 ± 3.4	6.8

Positive Control: amphotericin B (IC_50_ = 0.06 ± 0.02 μM); ND: not determinated; E1: hexane extract; E3F2: *n*-butanol fraction from E3 (methanol extract); E3F3: aqueous residue from E3 (methanol extract).

In this work, the antiviral activity against Herpes Simplex Virus type 1 (HSV-1, KOS strain) was also evaluated. Before the evaluation of the antiviral activity, the cytotoxic effects of the selected samples were investigated on VERO cells by MTT assay, and for each tested sample, a CC_50_ value was calculated. Of the 95 extracts and fractions tested, only the E3F2 fractions from the sponges *Haliclona* (*Halichoclona*) sp. and *P. citrina* showed antiviral activity (SI = 11.92 and, SI > 5, respectively).

In 2006, Silva and co-workers [20] performed an *in vitro* study on the antiherpes, anti-adenovirus and anti-rotavirus activities of marine sponges collected from the Brazilian coast, including *Haliclona* sp., *Polymastia janeirensis* and *T. ignis*. Of these, only the organic extract (methanol/toluene, 3:1 v/v) from *P. janeirensis* showed antiherpetic activity [20].

### 2.2. Marine Seaweeds

Only five out of 27 species from seaweeds assayed (Rhodophyta: *Digenea simplex*, *Laurencia dendroidea*, *Ochtodes secundiramea*, *Osmundaria obtusiloba*, andPhaeophyta: *Dictyota* sp.) showed weak growth inhibition zone (6 to 8 mm, (Table 6). Otherwise, *A. specifera*, *A. saldanhae*, *A. stellata*, *B. occidentalis*, *B. seaforthii*, *B. triquetrum*, *C. cervicornis*, *C. sertularioides*, *C. cupressoides*^a^, *C. cupressoides*^b^, *C. seminervis*, *D. delicatula*, *D. jolyana*, *G. caudata*, *G. cervicornis*, *G. cuneifólia*, *H. cenomyce*, *H. musciformis*, *P. gymnospora*, *Padina* sp*.*, *P. flagellifera*, *P. papillosa* and *Sargassum* sp. did not show any antimicrobial activity.

Antibacterial activity may vary according to the species division [33]. In this study species from the phylum Rhodophyta exhibited better results than species from Chlorophyta and Phaeophyceae. In this context, our results are in agreement with the findings of Padmakumar and Ayyakkannu [34]. Members of the family Rhodophyceae are prolific producers of acetogenins as well as mono-, sesqui-, di- and triterpenes, many of them halogenated [35]. Many articles have reported antimicrobial activity of halogenated sesquiterpenes and acetogenins derived from *Laurencia* species, especially for (−)-elatol, obtusol, (+)-obtusane, cartilagineol, and triquinane derivatives [36,37]. Studies performed with *Osmundaria* species are scarce, but some compounds so far described have potential antimicrobial activity, particularly the halogenated phenol derivatives, such as lanosol and sulfated oligobromophenols [38,39].

**Table 6:** Antibacterial and antifungal screening of marine seaweeds by disc diffusion method.

Species	Extracts	Bacterial and fungal strains				
		*S. aureus*	*E. faecalis*	*E. coli*	*P. aeruginosa*	*C. albicans*
*Dictyota* sp.	DS	+	+	−	+	−
*Digenea simplex*	DS	+	+	−	+	−
*Laurencia dendroidea*	FS	+	+	−	−	−
*Ochtodes secundiramea*	FS	+	−	−	−	−
*Osmundaria obtusiloba*	DS	−	−	−	++	−

(−): no activity; (+): 6–8 mm of inhibition zone; (++): 9–12 mm of inhibition zone; (+++): 13–16 mm of inhibition zone. Positive controls: *S. aureus*: oxacillin (1 µg) 18–24 mm; *E. faecalis*: ampicillin (10 µg) > 17 mm; *P. aeruginosa*: ceftazidime (30 µg) 22–29 mm; *E. coli*: ampicillin (10 µg) 16–22 mm; *C. albicans*: fluconazole (25 µg) > 19 mm; DS: extract obtained from dried seaweeds using CH_2_Cl_2_: MeOH (2:1); FS: extract from fresh seaweeds using Me_2_CO.

Regarding the antiprotozoal activity, of the 27 species assayed, six showed interesting activity against *L. braziliensis* and *T. cruzi*. Extracts from *Anadyomene saldanhae* (FS extract), *Caulerpa cupressoides*^(a)^ (FS extract), *Canistrocarpus cervicornis* (FS extract), *Dictyota* sp. (FS extract), *Ochtodes secundiramea* (FS extract), and *Padina* sp. (FS extract) showed promising results against *L. braziliensis* (87.9, 51.7, 85.9, 93.3, 99.7, and 80.9% growth inhibition, respectively). Only *Dictyota* sp. was effective against *T. cruzi* (60.4% growth inhibition) (Table 7). Otherwise, *B. triquetrum*, *C. sertularioides*, *C. cupressoides*^b^, *D. delicatula*, *G. caudata*, *H. cenomyce*, *H. musciformis*, *P. papillosa* and *Sargassum* sp., none antiprotozoal activity was detected.

As far as we are aware, there are no reports in the literature on the antiprotozoal activity for four of these seaweeds species (*A. saldanhae*, *C. cupressoides*^a^, *Padina* sp., and *O. secundiramea*). Concerning the activity of FS extract from the red seaweed *L. dendroidea* (= formerly *Laurencia obtusa*), only a weak (14.6%) antileishmanial activity against the promastigote forms of *L. braziliensis* was observed (Table 7). Furthermore, two species of seaweeds, *A. saldanhae* (SI = 12.3) and *Padina* sp. (SI = 7.5), were effective against *L. brasiliensis* amastigotes. Additionally, *C. cervicornis*, *C. cupressoides*^a^, *Dictyota* sp., and *O. secundiramea* were strongly cytotoxic for bone marrow macrophages (Table 8).

**Table 7:** Antiprotozoal activity expressed as growth inhibition (%) of extracts and fractions obtained from marine seaweeds.

Species	Extracts	*Leishmania braziliensis* (promastigotes)	*Trypanosoma cruzi* (epimastigotes)
*Anadyomene saldanhae*	FS	87.9	−
*Botryocladia occidentalis*	DS	20.7	−
*Bryothamnion* seaforthii	DS	33.5	−
*Canistrocarpus cervicornis*	FS	85.8	−
*Caulerpa cupressoides*	FS	51.7	−
*Dictyota* sp.	DS	93.3	60.4
*Digenea simplex*	DS	26	−
*Gracilaria caudata*	DS	9.3	−
*Grateloupia cuneifolia*	FS	35.2	15.9
	DS	37	23.98
*Laurencia dendroidea*	FS	14.6	−
*Ochtodes secundiramea*	FS	99.7	−
*Padina* sp.	FS	80.9	−
*Palisada flagellifera*	DS	21	−

Extracts and fractions concentration: 50 µg/mL; (−): no activity; DS: extract obtained from dried seaweeds using CH_2_Cl_2_/MeOH (2:1); FS: extract from fresh seaweeds using Me_2_CO.

**Table 8:** Effects of marine seaweeds extracts and fractions on *Leishmania brasiliensis* amastigotes in bone marrow macrophages from mice, and cytotoxicity on J774.G8 macrophage cell line.

Species	Samples	CC_50_ ± SD (µg/mL)	IC_50_ ± SD (µg/mL)	Selective index (CC_50_/IC_50_)
*Anadyomene saldanhae*	FS	294.2 ± 28.2	23.9±2.3	12.3
*Canistrocarpus cervicornis*	FS	>50	ND	ND
*Caulerpa cupressoides*^(a)^	FS	>50	ND	ND
*Dictyota* sp.	DS	>50	ND	ND
*Ochtodes secundiramea*	FS	>50	ND	ND
*Padina* sp.	FS	300.4 ± 28.5	40.2 ± 4.3	7.5

Positive Control: amphotericin B (IC_50_ = 0.06 ± 0.02 μM); ND: not determined; DS: extract obtained from dried seaweeds using CH_2_Cl_2_/MeOH (2:1); FS: extract from fresh seaweeds using Me_2_CO.

Previous studies performed by Veiga-Santos *et al*. [5] and Machado *et al*. [37] showed that lipophilic extracts from *L. dendroidea* collected from the southeastern coast of Brazil strongly inhibited the growth of *T. cruzi* and *L. amazonensis*. These results are not completely in agreement with our findings for *L. dendroidea* and this discrepancy may be due to the different geographic regions where this species was collected, as well as the seawater conditions.

Another study led by Santos and colleagues [4] found that lipophilic extracts from the brown seaweed *C. cervicornis* collected from the northeastern coast of Brazil also strongly inhibited the growth of *L. amazonensis*. From this species, a 4-acetoxydolastane diterpene was isolated, which demonstrated dose-dependent activity during 72 h of treatment, exhibiting IC_50_ values of 2.0, 12.0 and 4.0 μg/mL for promastigotes, axenic amastigotes and intracellular amastigotes of *L. amazonensis*, respectively.

Concerning antiviral activity, none of the species tested displayed any anti-HSV-1 activity. Although Soares and colleagues [25] reported the anti-HSV-1 activity for the red alga *L. dendroidea* collected from the coast of Rio de Janeiro, in our work this specie showed high cytotoxicity against VERO cells and none antiviral activity was detected.

To summarize, the present work reports the antimicrobial, antiprotozoal and antiviral evaluation of organic extracts from nine sponges, two octocorals, one ascidian, one bryozoan, and 27 seaweeds species, collected along the Brazilian coastline. Of a total of 95 extracts and fractions, 53 (56%) showed some anti-infective activity against *S. aureus*, *E. faecalis*, *P. aeruginosa*, *E. coli*, *C. albicans*, *L. braziliensis*, *T. cruzi*, and HSV-1.

Clearly, the marine invertebrates and seaweeds from the Brazilian coast could play an important part in the future control of the global infectious-disease burden. Although substantial progress has been made in identifying new biotechnological potential from these organisms, further chemical analysis and biological studies are required for investigating the mechanism of action, the chemical content as well as the potential use of these marine organisms extracts in the prevention of pathologies.

## 3. Experimental

### 3.1. Collection of the Marine Organisms

Marine invertebrates were collected in April/May 2011, at Xavier (27°36'39''S; 48°23'32''W), Arvoredo (27°17'00''S; 48°22'00''W) and Aranhas (27°29'12''S; 48°21'37''W) Islands, Florianópolis, Santa Catarina State, Brazil, at a depth of 9–17 m. They were immediately frozen and then lyophilized. For the identification, the sponges were submitted to dissociated spicule preparations, and thick sections were made according to Mothes-de-Moraes [40]. Voucher specimens were deposited in the Porifera Collection of the Museu de Ciências Naturais, Fundação Zoobotânica do Rio Grande do Sul (MCNPOR). Tunicate and bryozoa were deposited in the Invertebrate Collection of the Departamento de Ecologia e Zoologia, Universidade Federal de Santa Catarina (Table 1).

Seaweeds specimens (Rhodophyta, Pheophyceae, and Chlorophyta) were collected in the midlittoral zone of the southern and northeastern Brazilian coast, in August/October 2011 (Table 2). The epiphytic organisms from the seaweeds were manually cleaned immediately after collection, and air dried. The voucher specimens were deposited at the Herbarium of the Department of Botany at Universidade Federal de Santa Catarina, Brazil.

### 3.2. Preparation of the Extracts

**Figure 1:**
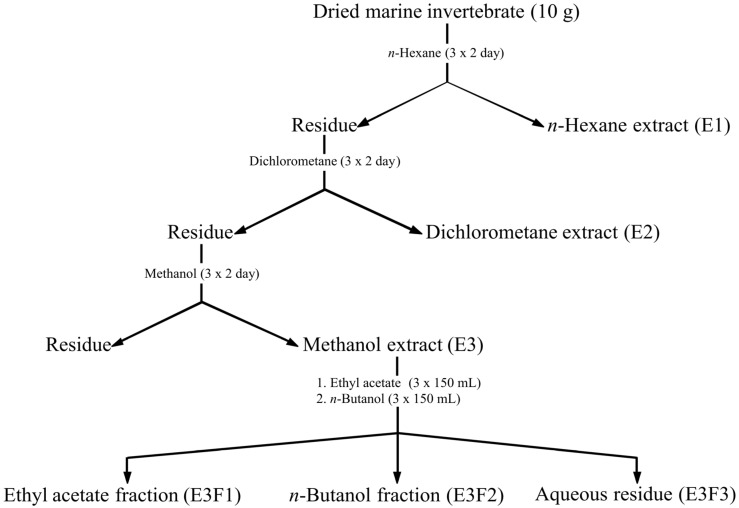
Procedure for obtaining the marine invertebrate extracts.

Organic extracts from marine invertebrates were prepared according to a standard procedure (Figure 1). Organic extracts from marine seaweeds were obtained using two distinct methods: CH_2_Cl_2_/MeOH (2:1) for dried seaweeds (DS extracts), and Me_2_CO for fresh seaweeds (FS extracts).

### 3.3. Antibacterial and Antifungal Assays

The microorganism strains tested were from the American Type Culture Collection (ATCC, Rockville, MD, USA): *Staphylococcus aureus* (ATCC 25923), *Enterococcus faecalis* (ATCC 29212), *Pseudomonas aeruginosa* (ATCC 27853), *Escherichia coli* (ATCC 25922), and *Candida albicans* (ATCC 10231).

The antibacterial and antifungal activities were evaluated by the disk diffusion method as previously described by de Oliveira *et al*. [41], with minor modifications. Briefly, filter paper disks (5 mm) were impregnated with 20 μl of the extracts or fractions solutions (100 mg/mL) and then placed on Muller-Hinton agar plates (HIMEDIA^®^), which were inoculated with the microorganisms according to the standard protocol described by the Clinical Laboratory Standard Institute [42]. The plates were incubated at 35 °C (± 1°C), and after 18 h, the diameters of the inhibition zones were measured. Filter-paper disks containing DMSO were used as negative control and no inhibition was observed. Standard antibiotic disks were selected according to the sensitivity of the microorganism tested: ampicillin (10 μg), oxacillin (1 μg), ceftazidime (30 μg) and fluconazole (25 μg) [43].

### 3.4. Antiprotozoal Activity

#### 3.4.1. Antileishmanial and Antitrypanosomal Activities

*Leishmania braziliensis* (MHOM/BR/96/LSC96-H3) promastigotes were grown at 26 °C in Schneider’s medium (Sigma Chemical Co., St. Louis, MO, USA) supplemented with 5% heat inactivated fetal bovine serum (FBS) and 2% urine. *Trypanosoma cruzi* (MHOM/BR/00/Y) epimastigotes were grown at 26 °C in Liver Infusion Tryptose (LIT) medium containing 10% FBS. Both parasite cultures were grown in 10 U/mL penicillin and 10 μg/mL of streptomycin (Gibco^®^). For the growth inhibition assays, *L. braziliensis* promastigotes or *T. cruzi* epimastigotes in the exponential phase of growth were harvested and washed twice in phosphate-buffered saline (PBS) by centrifugation at 1,500 × *g*. for 10 min. The parasites were counted in a Neubauer hemocytometer and seeded in 96-well microplates at 5.4 × 10^6^ (*T. cruzi*) or 3 × 10^6^ parasites/mL (*L. braziliensis*) in a final volume of 180 μL in LIT or Schneider´s medium, respectively. Parasites were incubated for 48 h at 26 °C in the presence of 20 μL of the samples (final concentration = 50 μg/mL). The standard drugs amphotericin B (Sigma) at 0.1 μM and benznidazole (Sigma) at 30 μM were used as positive controls and 1% DMSO was used as negative control. Parasite survival was assessed by the MTT assay [44]. The assays were carried out in triplicate, and the results were expressed as percentage of parasite growth inhibition.

#### 3.4.2. Activity against Intracellular Amastigotes of *T. cruzi* and *L. braziliensis* in Murine Macrophages

In this work, only the samples that showed parasite growth inhibition higher than 40% against the extracellular forms were analyzed through this methodology. Murine (Balb/C) bone marrow derived macrophages were differentiated for 7 days in 6 well plates, with Dulbecco’s Modified Eagle Medium (DMEM-Gibco) supplemented with HEPES (25 mM), penicillin (100 U/mL), streptomycin (100 μg/mL), FBS (10%) and 25% (v/v) supernatant of the murine fibroblast cell line L929 at 37 °C and 5% CO_2_, as described by Marim and co-workers [45], with minor modifications. Adherent cells were washed with PBS, trypsinized, counted in a Neubauer hemocytometer and concentration adjusted to 4.10^5^ cells/mL. Cell viability was assessed using Trypan Blue (0.04%). Next, 100 μl of cell suspension were seeded in 96 well plates and cultivated for 24 h at 37 °C. Thereafter, macrophages were infected with *L. braziliensis* axenic amastigotes (10 parasites/cell) for 3 h, at 34 °C and 5% CO_2_ or with VERO cell derived *T. cruzi* trypomastigotes (5 parasites/cell) for 4 h, at 37 °C and 5% CO_2_. Non-internalized parasites were removed by washing with PBS. After 24 h of incubation, 20 μL of the samples was added to the infected cell monolayers starting from 50 μg/mL and incubated for 48 h in 5% CO_2_ (34 °C for *L. braziliensis* and 37 °C for *T. cruzi*). The cells were washed with PBS, methanol fixed and Giemsa stained. The percentage of infected cells and the number of intracellular amastigotes were assessed using an Olympus IX70 optical inverted microscope, randomly counting 100 cells/well at a magnification of 400×. The reduction of the parasitic index was calculated as described elsewhere [46], and the 50% inhibitory concentration was calculated by linear least squares regression, using the software GraphPad Prism 5.0. Amphotericin B (0.2 μM) and benznidazole (15 μM) were used as positive controls. DMSO 1% was used as negative control. The experiments were carried out in triplicate and repeated at least twice.

#### 3.4.3. Cytotoxic Activity against J774.G8 Macrophage Cell Line

Murine J774.G8 phagocytic cells were seeded in 96 well plates with DMEM supplemented with HEPES (25 mM), penicillin (100 U/mL), streptomycin (100 μg/mL) and FBS (10%), and incubated for 72 h with the samples starting from 500 μg/mL. The assays were carried out in triplicate and cell viability was determined as described above for VERO cells. The CC_50_ was calculated by minimum square linear regression with the software GraphPad Prism 5.0.

### 3.5. Anti-HSV-1 Assay

#### 3.5.1. Virus and Cell Line

The cell line used (VERO-ATCC: CCL81) was grown in Eagle’s minimum essential medium (MEM; Cultilab, Campinas, Brazil) supplemented with 10% fetal bovine serum (FBS; Gibco, Carlsbad, CA, USA), 100 U/mL penicillin G, 100 µg/mL streptomycin and 25 µg/mL amphotericin B (Cultilab^®^). Cell cultures were maintained at 37 °C and 5% CO_2_. The HSV-1 (KOS strain, Faculty of Pharmacy, University of Rennes, France) was propagated in VERO cells. Viral stock was prepared, titrated based on plaque forming units (PFU), counted by the plaque assay as previously described [47] and stored at −80 °C.

#### 3.5.2. Cytotoxicity Assay

Confluent VERO cells were exposed to different concentrations of the samples for 72 h. After incubation, cell viability was assessed by the MTT [3-(4,5-dimethylthiazol-2,5-diphenyltetrazolium bromide] assay [44]. The assays were carried out in triplicate, and the results were expressed as the CC_50_, which was defined as the concentration that reduced cell viability by 50%, when compared to the untreated controls.

#### 3.5.3. Viral plaque Number Reduction Assay

This assay followed the procedures described by Kuo *et al*. [48], with minor modifications. Approximately 100 PFU of HSV-1 was adsorbed for 1 h at 37 °C on confluent VERO cells. Cultures were then overlaid with MEM containing 1.5% carboxymethylcellulose (CMC; Sigma) with or without different concentrations of the samples. After 72 h, the cells were fixed and stained with naphtol blue-black (Sigma), and the plaques were counted. The assays were carried out in triplicate, and the results were expressed as the IC_50_, which was defined as the concentration that reduced the number of viral plaques formed by 50%, when compared to the untreated controls. Acyclovir (Sigma) was used as a positive control.

## 4. Conclusions

In this work, we screened 95 different extracts and fractions from Brazilian marine seaweeds and invertebrates, for their potential anti-infective properties (antibacterial, antifungal, antiprotozoal and antiviral activities). The studies showed that invertebrates *Bugula neritina*, *Carijoa riisei*, *Dragmaxia anomala*, *Haliclona* (*Halichoclona*) sp. and *Petromica citrina* and seaweeds *Anadyomene saldanhae*, *Canistrocarpus cervicornis*, *Caulerpa cupressoides*, *Dictyota* sp., *Digenea simplex*, *Laurencia dendroidea*, *Ochtodes secundiramea* and* Osmundaria obtusiloba* showed some type/level of anti-infective property.

Moreover, this work also shows the importance of bioprospecting studies highlighting the importance of marine biodiversity as sources of potential natural compounds with pharmacological properties or biotechnological potential that could be used in the development of new drugs. All the active extracts deserve special attention in further studies to chemically characterize the bioactive compounds as well as more refined biological assays.
